# Efficacy and safety of the “Xingnao Kaiqiao” acupuncture technique via intradermal needling to treat postoperative gastrointestinal dysfunction of laparoscopic surgery: study protocol for a randomized controlled trial

**DOI:** 10.1186/s13063-017-2319-3

**Published:** 2017-11-28

**Authors:** Wenli Zhao, Jinting Li, Yuling Wang, Jing Liu, Ying Chen, Guang Zhao, Ye Zhao, Huaien Bu, Yiider Tseng, Xuemin Shi

**Affiliations:** 1 Graduate School, Tianjin University of Traditional Chinese Medicine, Tianjin, 300193 China; 2 Department of Neurology, Tianjin Nankai Hospital, Tianjin, 300100 China; 3 Department of Minimally Invasive Surgery, Tianjin Nankai Hospital, Tianjin, 300100 China; 4 Department of Chemical Engineering, University of Florida, Gainesville, Florida 32611 USA; 5 College of Traditional Chinese Medicine, Tianjin University of Traditional Chinese Medicine, Tianjin, 300193 China; 6 Department of Chemical Engineering, University of Florida, 1006 Center Drive, Gainesville, Florida 32611 USA; 7 Department of Acupuncture, First Affiliated Hospital, Tianjin University of Traditional Chinese Medicine, Tianjin, 300193 China

**Keywords:** *Xingnao Kaiqiao* acupuncture technique, Intradermal needling, Laparoscopic surgery, Postoperative gastrointestinal dysfunction, Ghrelin, Time of the first postoperative flatus

## Abstract

**Background:**

*Xingnao Kaiqiao* acupuncture involves needling of the *Neiguan* (PC6), *Renzhong* (DU26), and *Sanyinjiao* (SP6) acupoints. The technique has a significant clinical effect in many neurological diseases. In the present report, we have developed a protocol for a scientific trial to analyze whether *Xingnao Kaiqiao* can be used to treat gastrointestinal dysfunction after laparoscopic surgery. In this context, we intend to execute a double-blind, randomized controlled trial to assess the efficacy and safety of *Xingnao Kaiqiao* acupuncture via intradermal needling.

**Methods/design:**

This will be a single-center, double-blind, randomized controlled clinical trial. It has been designed on the basis of the Consolidated Standards of Reporting Trials (CONSORT 2010) guidelines and the Standards for Reporting Interventions in Controlled Trials of Acupuncture (STRICTA). The subjects will be recruited from among inpatients scheduled for laparoscopic surgery at the Department of Minimally Invasive Surgery, Tianjin Nankai Hospital, Tianjin, China. Using random numbers generated in SPSS 19.0, the recruited subjects will be allocated to either the “*Xingnao Kaiqiao*” group or the sham stimulation group. A specially appointed investigator will be in charge of the randomization. *Xingnao Kaiqiao* via intradermal needling (or sham needling) will be administered 6 h after laparoscopic surgery, and then every 12 h for a total of six sessions, each of which will last 3 min. The subjects will undergo their first evaluation shortly before the first treatment (6 h after laparoscopic surgery); evaluations will be repeated every 12 h until a total of seven evaluations have been completed. The primary outcome will be the time until the first postoperative flatus. The secondary outcomes will be: the time until the first postoperative defecation; levels of abdominal pain, abdominal distension, and nausea; blood ghrelin level; occurrence of vomiting; psychological status; and quality of life.

**Discussion:**

This upcoming randomized clinical trial was designed as a standardized method to assess the efficacy and safety of *Xingnao Kaiqiao* acupuncture using intradermal needles on PC6, DU26, and SP6 in the treatment of gastrointestinal dysfunction after laparoscopic surgery. We aim to provide evidence and thus improve the clinical application of this technique.

**Trial registration:**

Chinese Clinical Trial Registry, ChiCTR-IOR-17010763. Registered on 2 March 2017.

**Electronic supplementary material:**

The online version of this article (doi:10.1186/s13063-017-2319-3) contains supplementary material, which is available to authorized users.

## Background

Gastrointestinal dysfunction frequently occurs after abdominal surgery [1] and can lead to the accumulation of gastrointestinal secretions and gas. Thus, patients have trouble passing flatus and defecating, and this causes abdominal distension, abdominal pain, nausea, and vomiting. These symptoms affect postoperative recovery [2]. Severe cases can result in further complications, such as intestinal adhesions and obstructions, which affect patients’ quality of life. Currently, Western medicine treats postoperative gastrointestinal dysfunction using alvimopan. However, the side effects of this drug, which include nausea and vomiting, limit its widespread use [3]. In the case of abdominal surgery, successful postoperative rehabilitation would promote the early recovery of gastrointestinal function and minimize side effects [4].

Acupuncture has long been used in traditional Chinese medicine to treat gastrointestinal diseases, and considerable accumulated experience has highlighted its efficacy and advantages. Indeed, in 1997, the US National Institutes of Health published a consensus statement declaring that acupuncture can be an effective treatment for postoperative nausea and vomiting [5]. More recent research has shown that acupuncture promotes the recovery of gastrointestinal function after surgery [6–8]. These results have allowed practitioners to improve their use of acupuncture to treat postoperative gastrointestinal dysfunction.

Other research has shown that peripheral ghrelin is largely responsible for regulating gastrointestinal function, and that ghrelin in the central nervous system can influence gastrointestinal physiological activity [9]. High concentrations of ghrelin are found in the cell bodies and synapses of the hypothalamus, pituitary gland, and gastrointestinal vagus nerve [10, 11]. Therefore, it may be that acupuncture regulates gastrointestinal function by stimulating nerves, especially the vagus nerve. Specifically, the stimulation of acupoints may promote the release of ghrelin in both the central and peripheral nervous system. By way of comparison, one study reported that transcutaneous electrical stimulation of *Zusanli* (ST36) may alleviate limb muscle spasms caused by stroke-induced injury to motor neurons [12]. It is believed that this effect is mediated by neurotransmitters. Another study reported that acupuncture causes chain reactions in the peroneal and tibial branches of the sciatic nerve [13]. The same study showed that electrostimulation of ST36 stimulates the peroneal nerve, which then propagates the impulse through the sciatic nerve to the vagus nerve, causing the release of aromatic l-amino acid decarboxylase and leading to elevated levels of neurotransmitters. Similarly, electrostimulation of *Neiguan* (PC6) stimulates sensory fibers in the median nerve, which in turn excite the nucleus tractus solitarius of the vagus nerve causing neurotransmitter release [14]. In vagotomized patients, the central effects of ghrelin are blocked, indicating that vagus nerve stimulation promotes ghrelin release [15].

In 1972, Professor Xuemin Shi, a member of the Chinese Academy of Engineering, suggested that *Xingnao Kaiqiao* acupuncture (XNKQAT) can be used to treat encephalopathy. XNKQAT involves the use of needling acupoints to activate the brain’s nourishment of the liver and kidneys, as well as to promote the *flow of qi*. The principal acupoints of this technique are PC6, *Renzhong* (DU26), and *Sanyinjiao* (SP6). Since its inception 40 years ago, the application of XNKQAT has gradually been improved through clinical practice, clinical trials, and laboratory research, and it is used to treat a continuously expanding spectrum of diseases.

Neuroanatomical study of acupoints has shown that PC6 corresponds to the median nerve and that SP6 corresponds to the tibial nerve (a branch of the sciatic nerve), which, as described above, can excite the vagus nerve and promote the release of ghrelin. By the same token, DU26 corresponds to the buccal branches of the facial nerve and the second branch of the trigeminal nerve. The ends of the visceral afferent fibers of the vagus nerve and the somatic afferent fibers of the trigeminal nerve overlap in the spinal trigeminal nucleus, which only receives somatosensory input from the face and mouth. Hence, stimulation of DU26 causes nerve impulses to integrate at the spinal trigeminal nucleus, and this excitation can be transferred to the vagus nerve [16].

More importantly, in the XNKQAT, the manipulation standard of DU26 stimulation is “moistening of the eye”, that is, tear production. Relatedly, sensation in the lacrimal gland is mainly provided by the ends of parasympathetic fibers, almost all of which merge into the ophthalmic and maxillary branches of the trigeminal nerve to form the lacrimal nerve, which in turn branches into the lacrimal gland and the eyelid. Only intense, long-lasting DU26 stimulation can excite the sensory neurons of the lacrimal nucleus sufficiently to produce tears. Indeed, the appearance of tears in such cases indicates that the sensory neurons of the trigeminal nerve and the parasympathetic neurons of the facial nerve have been excited; the end result of such vagus nerve excitation is ghrelin release [17]. Therefore, the “refreshment and resuscitation” technique for treating premature ovarian failure takes fully into account meridians, acupoints, and manipulation standards. Based on application theory research into meridians, acupoints, and acupuncture treatments, we hypothesized that XNKQAT systematically improves gastrointestinal function by stimulating ghrelin release.

XNKQAT can be applied using different acupuncture methods, such as needling, acupressure, electroacupuncture, moxibustion, and others. Of these, intradermal needling is now commonly used; using this method, practitioners must accurately locate the correct acupoints to ensure that patients are treated effectively. However, few studies have reported successful treatment of postoperative gastrointestinal dysfunction using intradermal needling. Therefore, this article proposes a rigorously designed, double-blind, randomized controlled clinical trial to investigate the efficacy and safety of intradermal needling-based XNKQAT to treat gastrointestinal dysfunction after laparoscopic surgery. The trial is designed to provide high-quality scientific evidence that will promote the clinical application of XNKQAT.

A pilot trial has been conducted in six patients who underwent laparoscopic surgery. These subjects were randomly assigned to the “XN group” (XNKQAT) or the “SH group” (sham stimulation). The patients adhered strictly to the evaluation and treatment processes of this study, and we observed that XNKQAT via intradermal needling of PC6, DU26, and SP6 is effective in the treatment of gastrointestinal dysfunction after laparoscopic surgery. Specifically, the average time until the patients’ first postoperative flatus was shorter in the XN group (15.21 ± 2.89 h) than in the SH group (28.32 ± 4.31 h). This supports the hypothesis that XNKQAT via intradermal needling promotes the recovery of gastrointestinal function after laparoscopic surgery.

## Methods/design

### Design

**Fig. 1:**
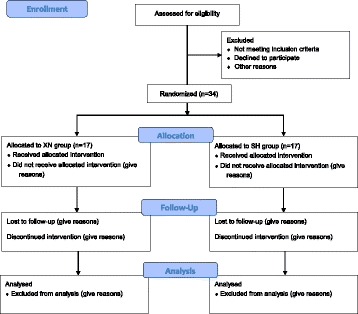
CONSORT 2010 flow diagram. *SH* sham, *XN Xingnao Kaiqiao* acupuncture technique

**Fig. 2:**
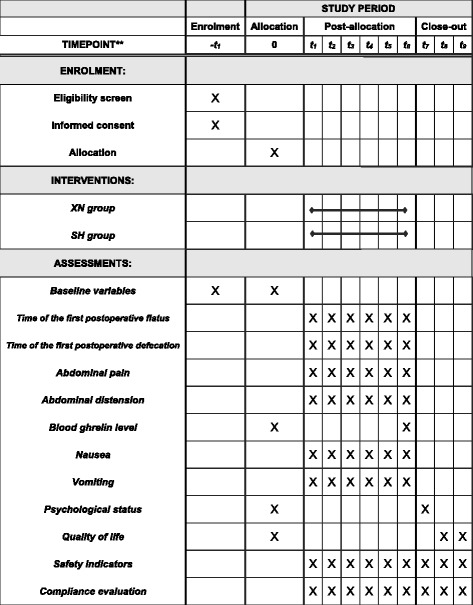
The schedule of enrollment, interventions, and assessments. *-t*_*1*_ the day before the surgery, *t*_*0*_ the day of the surgery, *t*_*1*_ *-t*_*6*_ the days in hospital after the surgery, *t*_*7*_ the day of the discharged, *t*_*8*_ 14 days after the discharged, *t*_*9*_ 30 days after the discharge, *SH* sham, *XN Xingnao Kaiqiao* acupuncture technique

This is a protocol for a double-blind, single-center, randomized controlled clinical trial that conforms to the Consolidated Standards of Reporting Trials (CONSORT 2010) guidelines (Fig. 1), as well as to the Standards for Reporting Interventions in Controlled Trials of Acupuncture (STRICTA) [18, 19]. We designed the trial on the basis of the Standard Protocol Items: Recommendations for Interventional Trials (SPIRIT) statement (Fig. 2) and its corresponding checklist (Additional file 1). Two groups will be tested in parallel and compared. The trial participants will be selected from among subjects who are to receive laparoscopic surgery in the Department of Minimally Invasive Surgery, Tianjin Nankai Hospital, Tianjin, China, between March and June 2017. The XNKQAT intradermal needling will be carried out by specialist nurses with at least 5 years of professional training in acupuncture.

Using random numbers generated in SPSS 19.0, we will allocate subjects to either the XN group or the SH group at a ratio of 1:1. To reduce selection bias, a specifically appointed investigator will be in charge of the random allocation and other confounding factors. The first XNKQAT treatment will take place 6 h after surgery and will be followed by another treatment every 12 h thereafter until a total of six treatments have been completed. Each treatment will last 3 min. The patients will subsequently attend three follow-up visits. In addition, seven evaluations will be performed to monitor outcomes related to the recovery of gastrointestinal function. The first such evaluation will take place immediately before the first treatment, 6 h after surgery. The evaluations will be repeated every 12 h thereafter until a total of seven evaluations have been completed.

### Study subjects

The study will recruit inpatients of the Department of Minimally Invasive Surgery, Tianjin Nankai Hospital, Tianjin, China, who are scheduled to undergo laparoscopic surgery between March and November 2017. Based on the six-subject pilot trial described above, which was performed in November 2016, the present trial will compare the time until first postoperative flatus between the two groups; we consider this the primary outcome in the recovery of gastrointestinal function. In addition, the pilot trial’s intervention was based on that of the present formal trial. The treatment in the pilot trial was needling (or sham needling) at PC6 on both forearms, SP6 on both ankles, and DU26; that is, at a total of five acupoints. Using the parameters α = 0.05 and power = 80%, and assuming a dropout rate of 15%, 34 participants will be required to participate in the full-scale study to ensure statistically meaningful results.

To ensure that the results are accurate, the present study will include strict inclusion, exclusion, and elimination criteria, as outlined below.

#### Inclusion criteria

To enroll in the trial, subjects must: 1) be aged 18–65 years; 2) be scheduled to undergo elective laparoscopic surgery under general anesthesia; 3) have no experience with intradermal needling therapy; 4) not be enrolled in any other clinical trials; 5) have no cognitive dysfunction, aphasia, mental illness, or other communication barriers; and 6) be able to sign an informed consent form.

#### Exclusion criteria

Subjects may not participate in the trial if they: 1) are pregnant or lactating; 2) have a history of abdominal surgery; 3) are to receive surgery other than laparoscopic surgery during their hospital stay; 4) have skin injuries at the selected acupoints; 5) are allergic to adhesive tape; 6) have severe gastrointestinal disease or chronic pain (e.g., chronic pelvic pain); 7) have been taking analgesic drugs for a long time or in the last 48 h; 8) have a serious cardiac, encephalic, hepatic, renal, or hematologic system disease; 9) have a history of substance (such as morphine) abuse, exercise-induced vomiting, diabetes, surgical vomiting, or obesity (body mass > 80 kg); or 10) have been bedridden for a long time.

#### Elimination criteria

Subjects will be eliminated from the trial if: 1) their laparoscopic surgery is changed to laparotomy; 2) they refuse to undergo the treatment for any reason; 3) serious syndromes, such as biliary fistula or peritonitis, arise during the treatment; 4) they transfer to another specialist for treatment; 5) they become comatose or die; 6) they are unable to attend the follow-ups for any reason; 7) they do not match the inclusion criteria and were accidentally included; and 8) they do not comply with the treatment or fail to provide information that may be important in the evaluation.

### Ethical issues

The Institutional Review Board (IRB) of Tianjin Nankai Hospital approved the trial on 10 February 2017 (IRB approval no. 2016-037P). After obtaining the IRB approval, we registered the trial on an authoritative registration platform of clinical trials (Chinese Clinical Trial Registry, ChiCTR-IOR-17010763) before the start of the study. The informed consent form (Additional file 2) was developed in accordance with the Declaration of Helsinki. All qualified patients can choose freely whether they wish to participate in the trial; those who do will be required to sign an informed consent form before enrolling.

### Randomization

Patients will be assigned in a 1:1 ratio to either the XN group or the SH group using random numbers generated in SPSS 19.0. To reduce selection bias and avoid other confounding factors, a specifically appointed investigator will be in charge of the randomization process. When recruiters identify a subject who meets the criteria for participation, they will obtain a sequence number for that subject from the researcher in charge of the randomization process.

To prevent the biases of the researchers from influencing the authenticity of the clinical findings, the subjects’ group allocations will be concealed from the recruiters and evaluators using the sealed envelope method. Briefly, the subjects’ allocation will be concealed inside sealed, opaque, serialized envelopes from the researchers responsible for recruitment and treatment evaluation. To ensure strict confidentiality, the envelopes will not be transparent, even under intense light. To prevent confusion around the group assignments, the subjects’ full names and birthdates will be written on the outside of their envelopes using carbon paper. The information on the sealed envelopes, and the details about the subjects, will be video-recorded, and the carbon copy of the label on the outside of the sealed envelope will then be attached to the group allocation card inside the envelope. Next, another researcher will check the videos to ensure the subjects’ envelopes are sealed. The entire process of opening the envelopes will also be video-recorded. The envelopes will be opened, and the assigned intervention type (XNKQAT or sham) will only be known after the respective subject has undergone all baseline evaluations.

### Blinding methods

This will be a double-blind trial. The subjects, evaluators, and statisticians will be unaware of the group assignment; that is, researchers evaluating the individual outcomes will be unaware of the group allocations, as will the statistician analyzing the data. It follows that the duties of the operators, data administrators, evaluators, and statistician will be mutually exclusive.

### Intervention

After laparoscopic surgery, the subjects will be asked to lie in the supine position without a pillow, and without eating or drinking. They will begin a liquid diet on the second day after surgery. The two groups will receive XNKQAT intradermal needling or sham stimulation 6 h after the surgery. Treatment will be repeated every 12 h thereafter until a total of six treatments have been administered. Each treatment will last for 3 min. Each time, the intradermal needles (or sham needles) will be attached to the surface of the acupoints. Six hours after surgery, immediately before the first treatment, the first evaluation will be carried out. The subjects will be evaluated every 12 h thereafter until a total of seven evaluations have taken place. Acupoints will be located in accordance with the World Health Organization Standard Acupuncture Point Locations in the Western Pacific Region [20]. The acupoints used will be PC6, DU26, and SP6. The nurse administering the treatment will stand next to the subject’s bed and help them to find a comfortable supine position.

### Treatment (XNKQAT) group

**Fig. 3:**
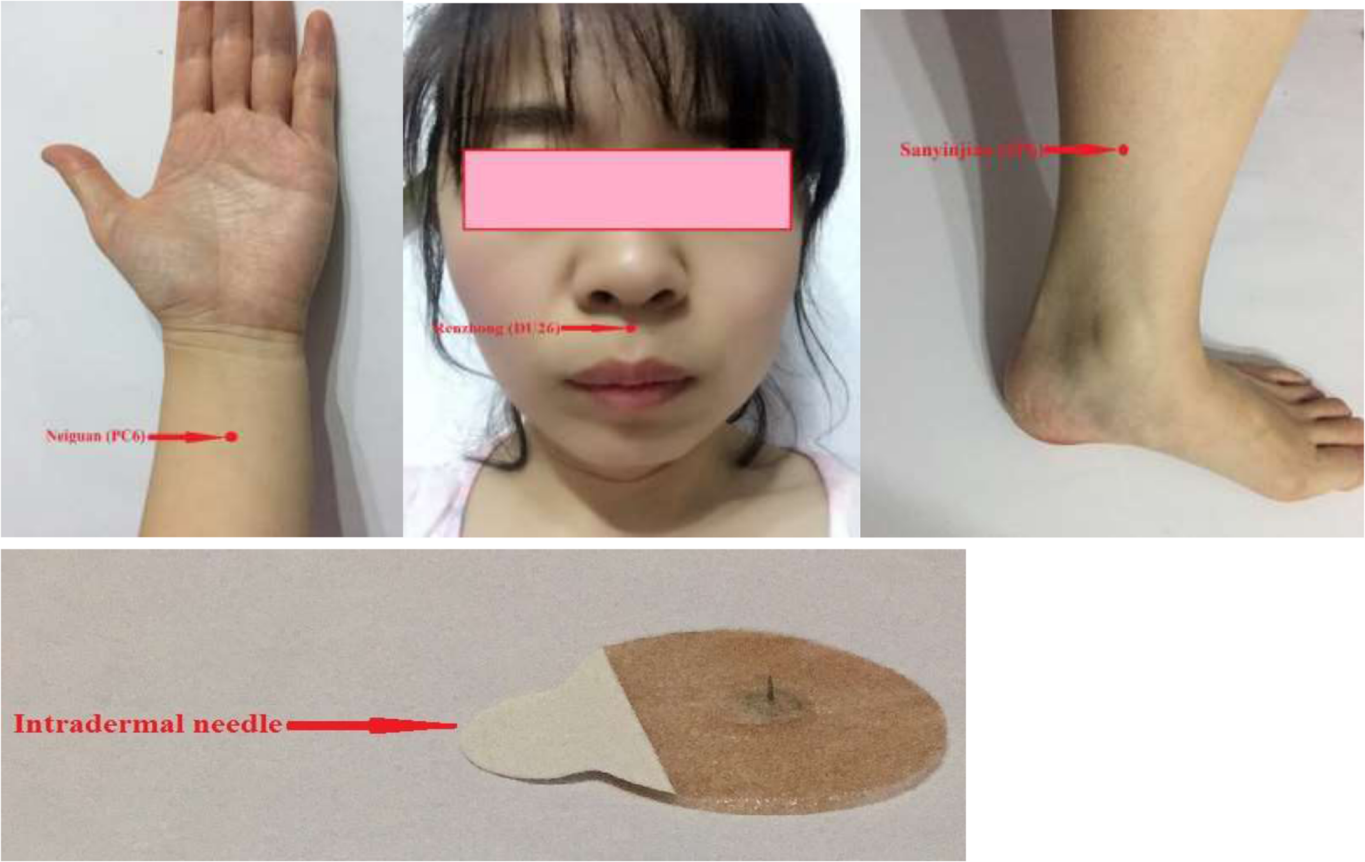
*Xingnao Kaiqiao* acupuncture technique and intradermal needle

**Fig. 4:**
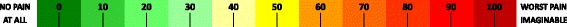
Visual analog scale

**Fig. 5:**
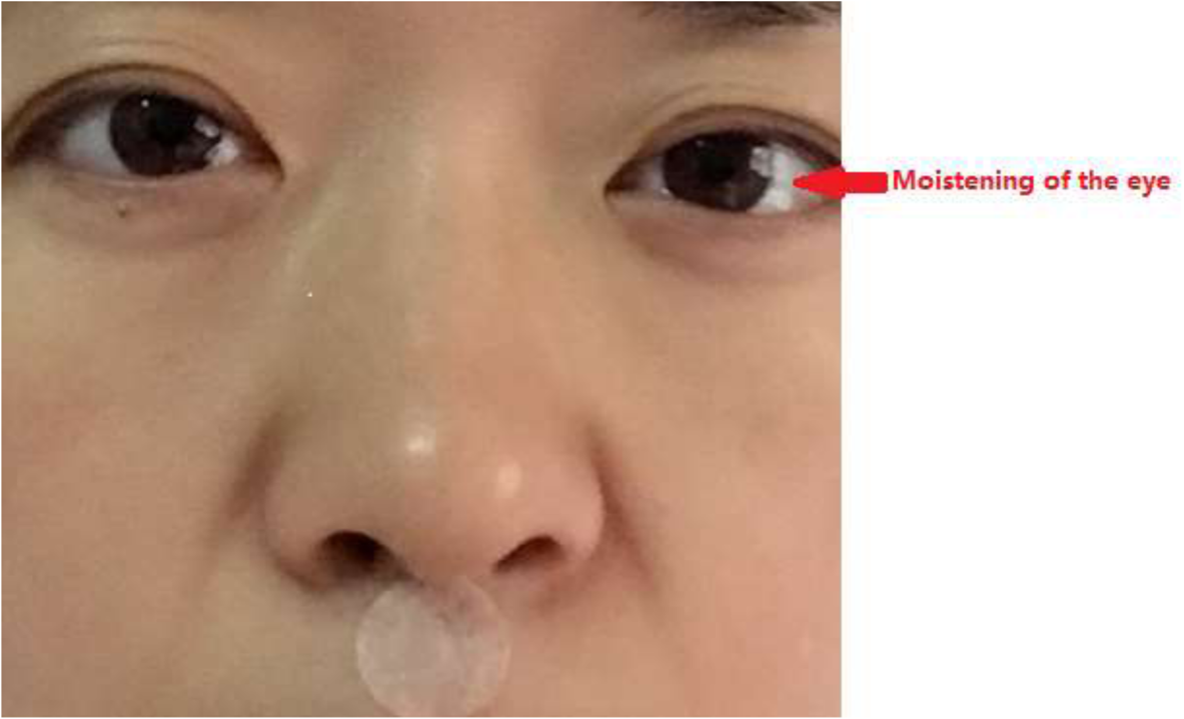
Moistening of the eye under the intradermal needling

This group of subjects will receive XNKQAT intradermal needling treatment at PC6 on both forearms, at SP6 on both ankles, and at DU26; that is, at a total of five acupoints (Fig. 3). After standard disinfection of the acupoints with 75% ethanol solution, the nurse will position the disposable medical intradermal needles (0.22 mm diameter × 1.5 mm length) perpendicular to the skin at the subject’s acupoints and then needle them into the skin. During the approximately 3-min treatment, the nurse will alternatively press the five intradermal needles for 15 s; a 20-s pause will occur between the repeated pressing intervals. To avoid injury to the skin or infection at the acupoints, the needles will not be twisted. The subject should feel a prick at the needled location. This study will use a visual analogue scale (VAS; Fig. 4) for pain [20] to determine the needle pressing force. Based on the VAS evaluation performed in the pilot trial, the pressing force will be limited to 40 mm. More importantly, in the XNKQAT, the manipulation standard of DU26 stimulation is “moistening of the eye,” that is, tear production (Fig. 5). Since the subjects will undergo a long pre-operative fasting period, excitation of the acupoints may trigger a hypoglycemic response. Therefore, it will be important to monitor the subjects’ faces during treatment to ensure they do not become pale or apathetic. If these signs occur, treatment will be stopped, the subjects’ blood glucose will be tested immediately, and appropriate management will be performed.

### Control (sham) group

In the SH group, the acupoints will be selected as described above. Based on the successful pilot trial, the nurse will use adhesive tape that is similar to intradermal needle tape at the acupoints described above. However, they will not use needles or press the points. The number of sessions, and the duration of treatment, will match the procedure in the XN group. To ensure that subjects in the SH group feel fully cared for, they will be provided with health-related information and support (complication awareness, dietary direction, exercise training, and psychotherapy), and they will be more verbally engaged than the treatment group.

### Outcome measures and subjects’ timelines

#### Outcome measures

##### Baseline information

Demographic information will be collected using a custom-made, standardized survey form that includes age, gender, educational background, occupation, weight, and height.

Surgical procedure information will be collected using a custom-made, form that collects information regarding clinical characteristics, including surgery and anesthesia duration, blood loss during surgery, infusion volume during surgery, and vital signs during surgery (body temperature, cardiac rate, breathing rate, and blood pressure).

##### Primary outcome

The time until the first postoperative flatus will be calculated, beginning at the time the subject returns to the ward after surgery. The subjects and their family members will be told to keep a record of the time until the first postoperative flatus:

Time until the first postoperative flatus = the moment of the first postoperative flatus – the moment when the subject returned to the ward after surgery

##### Secondary outcomes

*First postoperative defecation:* The time until the first postoperative defecation will be recorded, beginning at the time when the subject returns to the ward after surgery. The subjects and their family members will be instructed to record the time of the first postoperative defecation:

Time of the first postoperative defecation = the moment of the first postoperative defecation – the moment when the subject returned to the ward after surgery

*Abdominal pain:* A VAS will be used to estimate the level of the subjects’ postoperative abdominal pain [21]. The first assessment will be performed 6 h after the laparoscopic surgery, ahead of the first treatment. Thereafter, pain will be assessed every 12 h until a total of seven evaluations have been performed. The subjects and their families will assist in keeping the abdominal pain record.

*Abdominal distension:* A Likert scale will be used to evaluate the subjects’ abdominal distention after surgery [22]. The first assessment will be performed 6 h after laparoscopic surgery, ahead of the first treatment. Thereafter, distention will be assessed every 12 h until a total of seven evaluations have been performed. The subjects and their families will assist in keeping the abdominal distension record.

*Nausea:* A VAS scale will be used to evaluate the level of the subjects’ nausea [21]. The first assessment will be performed 6 h after laparoscopic surgery, ahead of the first treatment. Thereafter, nausea will be assessed every 12 h until a total of seven evaluations have been performed. The subjects and their families will assist in keeping the nausea record.

*Vomiting:* We will evaluate the number of times the subject vomits after surgery. The first assessment will be conducted 6 h after laparoscopic surgery, ahead of the first treatment. Thereafter, assessments will be performed every 12 h until a total of seven evaluations have been performed. The subjects and their families will assist in keeping the vomiting record.

*Ghrelin test:* Blood will be drawn for ghrelin testing once before surgery and again before discharge from the hospital. A total of 3 mL of blood will be collected from a vein in the morning, when the subject has an empty stomach. The blood sample will be allowed to sit for 30 min at room temperature and then be centrifuged at 3500 rpm for 10 min. The supernatant will be cryopreserved for ghrelin testing.

*Psychological status:* We will use the Hospital Anxiety and Depression Scale (HADS) to evaluate the subjects’ psychological status [23]. HADS has 14 items, of which seven evaluate depression and seven evaluate anxiety. To determine the severity of each condition, the depression and anxiety subscale scores are divided into categories of 0–7 points (asymptomatic), 8–10 points (symptoms suspected), and 11–21 points (symptoms indicated). The first assessment will be performed 6 h after laparoscopic surgery. Another HADS assessment will be performed on the day of discharge from the hospital.

*Quality of life:* We will use the Gastrointestinal Quality of Life Index (GIQLI) to evaluate the subjects’ quality of life [24]. The GIQLI was specifically designed to evaluate quality of life in subjects with gastrointestinal syndromes. It includes five aspects: subjective symptoms, physiological functions, daily life, social activities, and psychological/emotional state. There are 36 items in total, each adding 0–4 points to the score. Thus, the highest possible score is 144 points; higher scores indicate better quality of life. A normal score is in the range 121–125. The GIQLI will be evaluated three times. The first assessment will occur 6 h after the laparoscopic surgery; the second will occur on the day of discharge from the hospital, and the third will occur 1 month after discharge from the hospital.

##### Safety indicators

An adverse event is defined as at least four subjects suffering from the same symptom—such as local skin pain, itching, ulcers, bleeding, dizziness, or hypoglycemia—during the treatment and the follow-up period. Regardless of whether the unfavorable reactions or adverse events are related to the treatment, the researchers responsible for the treatment of the subjects will keep detailed records. Based on a statistical analysis of these events, XNKQAT safety will be evaluated.

If adverse events occur, the researchers will select an appropriate treatment method until the condition is stabilized. After the subject’s condition returns to normal, the researchers will decide whether further observation is required. If serious adverse events occur, the investigators will immediately report to the IRB and manage the subject’s condition appropriately.

##### Subjects’ timeline

One day before a given subject’s laparoscopic surgery, the investigators will decide whether to include them in the trial. This will be considered day 0 of the experiment. The subjects will receive their first postoperative intervention 6 h after surgery. Thereafter, interventions will be repeated every 12 h until a total of six interventions have taken place. The first evaluation will occur on day 0. Evaluations will then take place after each intervention, for a total of seven evaluations. For an overview of the recruitment timeline, interventions, and subject assessment, see Fig. 2.

### Data collection

For reasons of convenience, we will consolidate all subjects’ scores, observation times, adverse event records, and safety assessments into a single case report form (CRF). We will require the researchers to fill out the CRFs immediately and accurately.

### Quality control

#### Quality control system

We will establish a two-level quality assurance system. The first level will consist of quality inspection. For this purpose, the project leader (PL) will appoint a quality inspector, who will check the original data records, data reports, and adverse event logs using a quality assurance list. This person will also investigate any possible violations of protocol. The PL will immediately select an appropriate course of action if any quality problems arise. The second level will consist of quality monitoring. The clinical research monitor will monitor the medical practitioners to ensure they have an appropriate understanding of the research program, check that the processes are being correctly implemented, and ensure that all research data, reports, and CRFs are true, accurate, complete, and consistent with the actual data.

#### Quality control specifications

1) We will analyze potential confounding factors to decrease bias in the research results. 2) We will strictly execute the trial protocol and adhere to the standards of the treatment methods. 3) All investigators are certified doctors or nurses with professional acupuncture training. 4) We will strictly implement the randomization program that assigns subjects to two groups. 5) Researchers familiar with the scales will design the protocol regarding scoring methods, evaluation times, and evaluation forms. They will also standardize the way in which questions relate to the scales. The evaluators will adhere strictly to these principles when carrying out clinical evaluations. 6) The investigators will closely observe the included subjects and fill out the CRFs in accordance with the instructions. The data must be recorded diligently and completely. After attaching the data inspection report, no investigator will be permitted to change the original data. If any change is made, a detailed explanation must be provided and signed by the modifier. 7) The devices to be used in the trial will be purchased in one batch for consistency and reliability of data collection and research outcomes. 8) Subject compliance will be ensured.

### Sample size estimation and statistical analysis

#### Sample size estimation

The trial will test two groups in parallel. The following formula was used to estimate the required sample size:

$$ n=\frac{{\left({Z}_{1-\alpha /2}+{Z}_{1-\beta}\right)}^2\kern0.5em \times \kern0.5em \left({\sigma}_1^2+{\sigma}_2^2\right)}{\delta^2} $$

In this formula, *n* is the number of samples in each group, σ is the standard deviation, and δ represents the difference with clinical significance between two groups. Using the parameters *α* = 0.05 and power = 80%, the corresponding Z values for a bilateral test are Z_1–α/2_ = 1.96, Z_1–β_ = 0.84. In the November 2016 pilot trial, the average times until the first postoperative flatus in the XN and SH groups were S_1_ = 2.89 h and S_2_ = 4.31 h, respectively. Based on this clinical experience, we defined 4 h as a clinically significant difference in time until first postoperative flatus between the two groups. By substituting these values into the formula above, we obtained the parameter *n* = 13.19. Rounding up to the next whole number, the number of samples in each group should be 14. Assuming a loss rate of 15%, the final number of samples in each group will be 17. Hence, the total number of subjects in the present study will be 34.

#### Statistical analysis

##### Analysis procedures

*Sample distribution:* We will describe the size and dropout rate of each dataset. Detailed reasons for any dropouts will be provided.

*Balanced comparison:* We will compare the general subject data and then evaluate comparability between the two groups.

*Efficacy analysis:* We will use the time until the first postoperative flatus as a primary outcome to assess the efficacy of XNKQAT by intradermal needling in the treatment of gastrointestinal dysfunction after laparoscopic surgery. We will calculate the average and standard deviation to describe the outcome and use an independent-samples *t* test to compare the two groups. Eight secondary outcomes (time until first postoperative defecation; level of abdominal pain, abdominal distension, and nausea; blood ghrelin level; vomiting; psychological status; and quality of life) will be compared between the two groups to determine the efficacy of XNKQAT intradermal needling. The mean and standard deviations of these parameters will be reported. To record the subjects’ psychological status, we will record their scores and determine their classification based on the standards mentioned above. To determine the time until the first postoperative defecation, we will use the same method as the time until the first postoperative flatus. To analyze abdominal pain, abdominal distension, nausea, vomiting, ghrelin level, and quality of life, we will use repeated measures analysis of variance (RMANOVA). Before RMANOVA, we will carry out a test of sphericity to ensure that the data are suitable for this method. If the data do not meet spherical symmetry requirements, we will use the ε correction factor to correct the degrees of freedom before applying RMANOVA. The Mann–Whitney *U* test or Spearman’s Rank correlation coefficient tests will be used to analyze the categorization standards for psychological status.

*Safety analysis:* According to the definition of adverse events, specific adverse events will be listed, along with their severity level, causes, and explanations. The number of adverse events, and the adverse event rate, will be described statistically. If they need to be compared between groups, the χ^2^ test or Fisher’s exact test will be used.

#### Statistical analysis methods

The data will be analyzed according to the intention-to-treat (ITT) principle; that is, all subjects who are initially included in one of the two groups will be considered in the analysis. The analysis of efficacy and safety indicators will be carried out per-protocol (PP) and will include all subjects who complete the whole trial process. The quantitative variables of the research data will be statistically described using $\overline{\mathrm{X}}\kern0.5em \pm \kern0.5em \mathrm{SD}$. A record of qualitative variables will be kept using the number of cases in each category. To compare quantitative variables, if the data come from repeated measurements, the RMANOVA method will be used, and sphericity testing will be used beforehand to determine whether a correction is needed. Qualitative data will be analyzed using the Mann–Whitney *U* test or Spearman’s rank correlation. The χ^2^ test or Fisher’s exact test will be used to compare the occurrence rates of countable data. Statistical testing will be two-tailed, with *P* values < 0.05 considered significant. SPSS 19.0 software will be used for all statistical calculations.

## Discussion

Since postoperative gastrointestinal dysfunction seriously affects prognosis after abdominal surgery, clinicians and researchers must find treatments that promote early recovery of gastrointestinal function. Acupuncture is an important component of traditional Chinese medicine. It is technically simple to perform and easy to teach. Some research has indicated that acupuncture promotes postoperative recovery of gastrointestinal function [25, 26]. However, few high-quality studies support the technique in this regard. Therefore, to improve treatment, we conceived of the clinical method used in the pilot trial. In the proposed study, we will use XNKQAT intradermal needling to treat gastrointestinal dysfunction after laparoscopic surgery. Furthermore, since the time until the first postoperative flatus has clinical significance in postoperative recovery of gastrointestinal function [27], we will use it as the primary outcome in the trial.

A lot of studies have focused on acupuncture, its therapeutic effects, and its procedures, which are based on thousands of years of empirical practice. Furthermore, acupuncture is now used worldwide to treat diseases, and many studies have applied advanced molecular biology methods to study acupuncture. However, since traditional Chinese medicine is based on a different philosophy from translational medicine, it is difficult to incorporate acupuncture into treatment regimens. Nevertheless, recent advances in neuroendocrinology and immunology have allowed a better understanding of acupuncture. From a translational medicine perspective, we herein present a clinical trial protocol that adheres to scientific standards to evaluate the safety and efficacy of XNKQAT in the treatment of postoperative gastrointestinal dysfunction. The results of the trial will determine whether this treatment should be more widely applied in clinical practice. In addition, we intend to use this work to emphasize that the effects of acupoint stimulation on the human endocrine system must be mapped; this will enable further integration of acupuncture into current scientific practice. Herein, we present a promising new approach to the fusion of acupuncture and mainstream medicine, and we use XNKQAT as an example.

### Trial status

Recruitment for this trial is currently ongoing.

## Additional files

Additional file 1: SPIRIT 2013 checklist. (DOC 122 kb)

Additional file 2: Informed consent form. (DOCX 14 kb)

## Abbreviations

CRF: Case report form
DU26: *Renzhong*
GIQLI: Gastrointestinal Quality of Life Index
HADS: Hospital Anxiety and Depression Scale
IRB: Institutional Review Board
ITT: Intention-to-treat
PC6: *Neiguan*
PL: Project leader
PP: Per-protocol
RMANOVA: Repeated measures analysis of variance
SP6: *Sanyinjiao*
ST36: *Zusanli*
VAS: Visual analogue scale
XNKQAT: *Xingnao Kaiqiao* acupuncture technique
