# Supplementary material for: Efficacy and safety of the “Xingnao Kaiqiao” acupuncture technique via intradermal needling to treat postoperative gastrointestinal dysfunction of laparoscopic surgery: study protocol for a randomized controlled trial
Source: Trials. 2017 Nov 28;18:567. doi: 10.1186/s13063-017-2319-3 (PMC5704354; doi:10.1186/s13063-017-2319-3)
Supplement: Supplementary file 2 — Informed consent form. (DOCX 14 kb) [file 13063_2017_2319_MOESM2_ESM.docx]

**Informed Consent Form**

**Clinical trial’s name:** Efficacy and safety of the “Xingnao Kaiqiao” acupuncture technique via intradermal needling to treat postoperative gastrointestinal dysfunction of laparoscopic surgery: study protocol for a randomized controlled trial

**Primary sponsor:** Tianjin Nankai Hospital

**I volunteer to participate in this trial, and I have been informed:**

The trial will be based on the principle of ***Helsinki declaration***. And we strictly protect your privacy, that is, all the data of this trial will be kept strictly confidential, and your personal information will not appear in the Research Report or related publications. This trial has been reviewed by the medical ethics committee, which is considered to be safe and ethical, and the relevant research work has strictly complied with the spirit of the ***Helsinki declaration***.

1. I have learned the purpose and character of the trial through the researchers.
2. "Xingnao Kaiqiao" and intradermal needling is safe in treating postoperative gastrointestinal dysfunction of laparoscopic surgery.
3. This trial is used to observe the therapeutic safety and effectiveness of "Xingnao Kaiqiao" acupuncture technique via intradermal needling in treating postoperative gastrointestinal dysfunction of laparoscopic surgery in terms of a randomized controlled trial method.
4. I have learned that personal data in this trial will be strictly confidential and will not be disclosed.
5. I am aware of the significance, purpose and specific methods of this clinical trial, and also the expected outcomes, possible benefits and possible risks.
6. I know that I have the right to ask questions at any time, and have the right to decide at any time to quit the trial without any discrimination or retaliation, but still continue to be related to medical care.

I volunteer to participate in this trial. I have been informed and understand the trial’s situation. I confirm that there are plenty of time to consider and all difficult issues have been satisfactorily answered. I agree to participate the trial and try to ensure compliance.

**Name of the subject (signature):**

**Subject’s phone number:**

**Date:**
